# Supplementary material for: A New Face of the Old Gene: Deletion of the PssA, Encoding Monotopic Inner Membrane Phosphoglycosyl Transferase in Rhizobium leguminosarum, Leads to Diverse Phenotypes That Could Be Attributable to Downstream Effects of the Lack of Exopolysaccharide
Source: Int J Mol Sci. 2023 Jan 5;24(2):1035. doi: 10.3390/ijms24021035 (PMC9860679; doi:10.3390/ijms24021035)
Supplement: Supplementary file 1 [file ijms-24-01035-s001.zip › Supplementary Data for PssA final.pdf]

Supplementary Data for

A New Face of the Old Gene: Deletion of the PssA, Encoding Monotopic Inner Membrane Phosphoglycosyl Transferase in *Rhizobium leguminosarum*, Leads to Diverse Phenotypes That Could Be Attributable to Downstream Effects of the Lack of Exopolysaccharide

Małgorzata Marczak *, Kamil Żebracki †, Piotr Koper †, Aleksandra Horbowicz, Magdalena Wójcik and Andrzej Mazur *

Department of Genetics and Microbiology, Institute of Biological Sciences, Maria Curie-Skłodowska University, Akademicka 19 St., 20-033 Lublin, Poland

* Correspondence: malgorzata.marczak@mail.umcs.pl (M.M.); andrzej.mazur@mail.umcs.pl (A.M.)

† These authors contributed equally to this work.

This PDF file includes:
Supplementary Tables S1 to S7
Supplementary Figures S1 to S6
Supplementary File S1. Optimization of high-quality RNA extraction protocol from *Rhizobium leguminosarum* (includes: Table S8; Figures S7 And S8)
Note: Tables S3–S7 were provided as a Supplementary Excel files

Supplementary Table S1. Strains and plasmids used in this work

Strains	Relevant properties	References
Escherichia coli		
DH5α	F⁻ φ80*lacZ*ΔM15 Δ(*lacZYA–argF*) U169 *deoR recA1 endA1 hsdR17* (r_k^-, m_k^+) *phoA supE44* λ⁻ *thi*-1 *gyrA96 relA1*, high efficiency transformation strain	[1]
S17-1	294 derivative, RP4-2-Tc::Mu-Km::Tn7 chromosomally integrated, mobilizing donor strain	[2]
DHM1	Reporter strain for BTH system; F- *glnV*44(AS) *recA*1 *endA gyrA*96 *thi*-1 *hsdR*17 *spoT*1 *rfbD*1 *cya*-854	[3]
C41(DE3)	*F– ompT hsdSB (rB- mB-) gal dcm (DE3)*	Novagen
Rhizobium leguminosarum bv. *trifolii*		
RtTA1	wild type strain, Str^R, Rif^R	[4]
Δ*pssA*(Gm^R)	RtTA1 Δ*pssA*::Gm^R	This work
Δ*pssA*[pCM157]	RtTA1 Δ*pssA* carrying pCM157 *cre* expressing vector	This work
Δ*pssA*	RtTA1 Δ*pssA*	This work
Δ*pssA*(*pssA*)	RtTA1 Δ*pssA* carrying pBKpssA-C	This work
Δ*pssA*(*pssA*his)	RtTA1 Δ*pssA* carrying pBKpssA-His6	This work
Plasmids	Relevant properties	References
pCM351	*ori* ColE1, *oriT*, Ap^R, Gm^R, Tc^R, allelic exchange vector	[5]
pCM157	*ori* IncP, *oriT*, Tc^R, *cre* expression vector	[5]
pBBR1-MCS2	pBBR1 *rep*, *mob*, *lacZα* multi cloning site, Km^R, broad-host-range cloning vector	[6]
pET-30c(+)	*ori* pBR322, Km^R, *lacI*, 6xHis-tag, S-tag	Novagen
pET30-pssA	pET-30c(+) with 788 bp NdeI-KpnI fragment comprising *pssA* CDS	This work
pCGpssA-D	pCM351 with 645 bp ApaI–SacI fragment comprising 645 bp downstream of *pssA*	This wok
pCGpssA-UD	pCGpssA-D with 584 bp KpnI–NotI fragment comprising last 7 bp of *pssB* and 577 bp of *pssB–pssA* intergenic region	This wok
pBKpssA-C	pBBR1MCS-2 with 1739 bp KpnI–XbaI fragment comprising *pssB–pssA* intergenic region, *pssA*, and 370 bp downstream of *pssA*	This work
pBKpssA-His6	pBBR1MCS-2 with 1387 bp KpnI–BamHI fragment comprising *pssB–pssA* intergenic region and *pssA* without stop codon, equipped with His6-tag coding sequence and TAA stop codon	This work
pUT18	*cyaA*T18, Ap^r	[3]
pUT18C	*cyaA*T18, Ap^r	[3]
pKNT25	*cyaA*T25, Km^r	[3]
pKT25	*cyaA*T25, Km^r	[3]

pUT18C-zip	Two-hybrid control plasmid, Ap^r	[3]
pKT25-zip	Two-hybrid control plasmid, Km^r	[3]
pUT18-*pssADECSFGHIJ*	pUT18 with p*ssADECSFGHIJ* gene	[7]
pUT18-*pssTL*	pUT18 with p*ssTL*	[8]
pUT18-*pssP2*	pUT18 with p*ssP2*	[9]
pUT18C-*pssADECSFGHIJ*	pUT18C with p*ssADECSFGHIJ* gene	[7]
pUT18C-*pssPTL*	pUT18C with p*ssPTL*	[8]
pUT18C-*pssP2*	pUT18C with p*ssP2*	[9]
pKT25-*pssADECSFGHIJ*	pKT25 with p*ssADECSFGHIJ* gene	[7]
pKT25-*pssPTL*	pKT25 with p*ssPTL*	[8]
pKT25-*pssP2*	pKT25 with p*ssP2*	[9]
pKNT25-*pssADECSFGHIJ*	pKNT25 with p*ssADECSFGHIJ* gene	[7]
pPLE01	pBluescript II SK(+) with *phoAlacZ*α from pMA632; Ap^r	[10]
pPLE01-A85[a]	pPLE01 with 270 bp fragment spanning the 5′ end of the *pssA* gene cloned into the XbaI-BamHI sites	This work
pPLE01-A180[a]	pPLE01 with 555 bp fragment spanning the 5′ end of the *pssA* gene cloned into the XbaI-BamHI sites	This work
pPLE01-A263[a]	pPLE01 with 828 bp fragment spanning the entire *pssA* gene cloned into the XbaI-BamHI sites	This work
pPLE01-T201[a]	pPLE01 with 618 bp fragment spanning the 5′ end of the *pssT* gene cloned into the SacI-BamHI sites	This work
pPLE01-T243[a]	pPLE01 with 744 nt fragment spanning the 5′ end of the *pssT* gene cloned into the SacI-BamHI sites	This work

[a] PCR products included the 15-bp region in front of the ATG (TTG) codon, containing the ribosomal binding site (RBS). In plasmid names, the letters A and T denote the *pssA* or *pssT* gene, respectively

Supplementary Table S2. List of primers used in this work

Names	Sequences (5′–3′)[a]	References	Application
pssA-U_FwKpn	aaaggtaccGGCGTGACCATCCTTAGCATCG	This work	amplification of genomic fragments for Δ*pssA* mutant construction
pssA-U_RvNot	aagcggccgcCAGGGCTGCACTTAGGTCTTGGT	This work	
pssA-D_FwApa	aagggcccGCTTTAAGCTTTGTGAGAAAGGGC	This work	
pssA-D_RvSac	agagctcCTCTGGCGCAAACAACCGATC	This work	
pssA-C_FwKpn	aaaggtaccCCATCCTTAGCATCGCTGAATACC	This work	amplification of genomic fragments for Δ*pssA* mutant complementation
pssA-C_RvXba	aatctagaCAGCGACACATACGCGACTTCCT	This work	
pssA-His6_RvBam	aaggatccttaatgatgatgatgatggtgGAAGCCTTTACC ACCGGTCAGCTC	This work	
pCMFw1	GGGTTCCGCGCACATTTC	[11]	validation of cloning and sequencing of the pCM351 derivatives
pCMRv1	GCTGCGTTCGGTCAAGGT	[11]	
pCMFw2	CCTAACAATTCGTTCAAGCCGA	[11]	
pCMRv2	CGCGCGAACGACATGGAG	[11]	
pssA-O_Fw	CAATCCTGCCTTGATTTTGTCACAT	This work	confirmation of successful *pssA* mutagenesis
pssA-O_Rv	CGCAGGAATATTATTGAACGCACC	This work	
pssA-I_Fw	GAGCCTGAAGATCCAGACCCCTGT	This work	
pssA-I_Rv	AATGATGCGCATGTCCATCCAG	This work	
M13pUCf	CCCAGTCACGAAGTTGTAAAACG	Universal primer	validation of cloning and sequencing of the pBBR1-MCS2 derivatives
M13pUCr	AGCGGATAACAATTTCACACAGG	Universal primer	
PssABamFw	aaaggatccTGACAGGGTTAACCATTGATCG	This work	amplification of genomic fragment to construct pET30-pssA
PssAHinRv	aaaaagcttAGAAGCCTTTACCACCG	This work	
pET_ter	TGCTAGTTATTGCTCAGCGG	Universal primer	validation of cloning and sequencing of the pET-30c(+) derivatives
pET_up	GATGCGTCCGGCGTAGA	Universal primer	
repCa-RT_Fw	GGGATTGCCGCCTACCGTGACA	This work	confirmation of effective elimination of gDNA from the total RNA preparation of RtTA1
repCa-RT_Rv	TGGCTGGCCTTGAGTTCCCTTTGA	This work	
pssG-RT_Fw	GGTTCTGGGCCATCTGGAAGTA	This work	
pssF-RT_Rv	CCTATGGCGAGCGGATCCTG	This work	
AFwrbspLEOXbaI	aaatctagaAAGAGCAGCCCTGGTGAC	This work	amplification of genomic fragments to construct pPLE01 derivatives
ARv85pLEOBamHI	aaaggatccGGCGAGGACGAGGAGGG	This work	
ARv180pLEOBamHI	aaaggatccTGCATGGCAACGCGGGC	This work	
ARv263pLEOBamHI	aaaggatccGAAGCCTTTACCACCGGT	This work	
pssTFWrbspLE01SacI	aaagagctcTCTAAGAGGTTGCAATGGCTTTG	This work	
pssTA201RVnew	aaaggatccCGCGGTCAGGCTGTCGA	This work	
pssTA243RVnew	aaaggatccCGCGTTTCGGCCCGGCT	This work	
phoAlacZseq	CATCCCATCGCCAATCAGCA	This work	validation of cloning and sequencing of the pPLE01 derivatives

[a] introduced restriction sites are underlined

Supplementary Table S3. RNA-seq reads QC and filtering statistics

Supplementary Table S4. RNA-seq WT vs. Δ*pssA* raw data set summary

Supplementary Table S5. Differentially expressed genes (DEGs) log_2fold change values

Supplementary Table S6. DEGs rate in individual RtTA1 replicons

Supplementary Table S7. Comparison of DEGs detected in the Δ*pssA* transcriptome and proteins included previously into PssA stimulon in proteomic analysis by Geurreiro et al. [12]

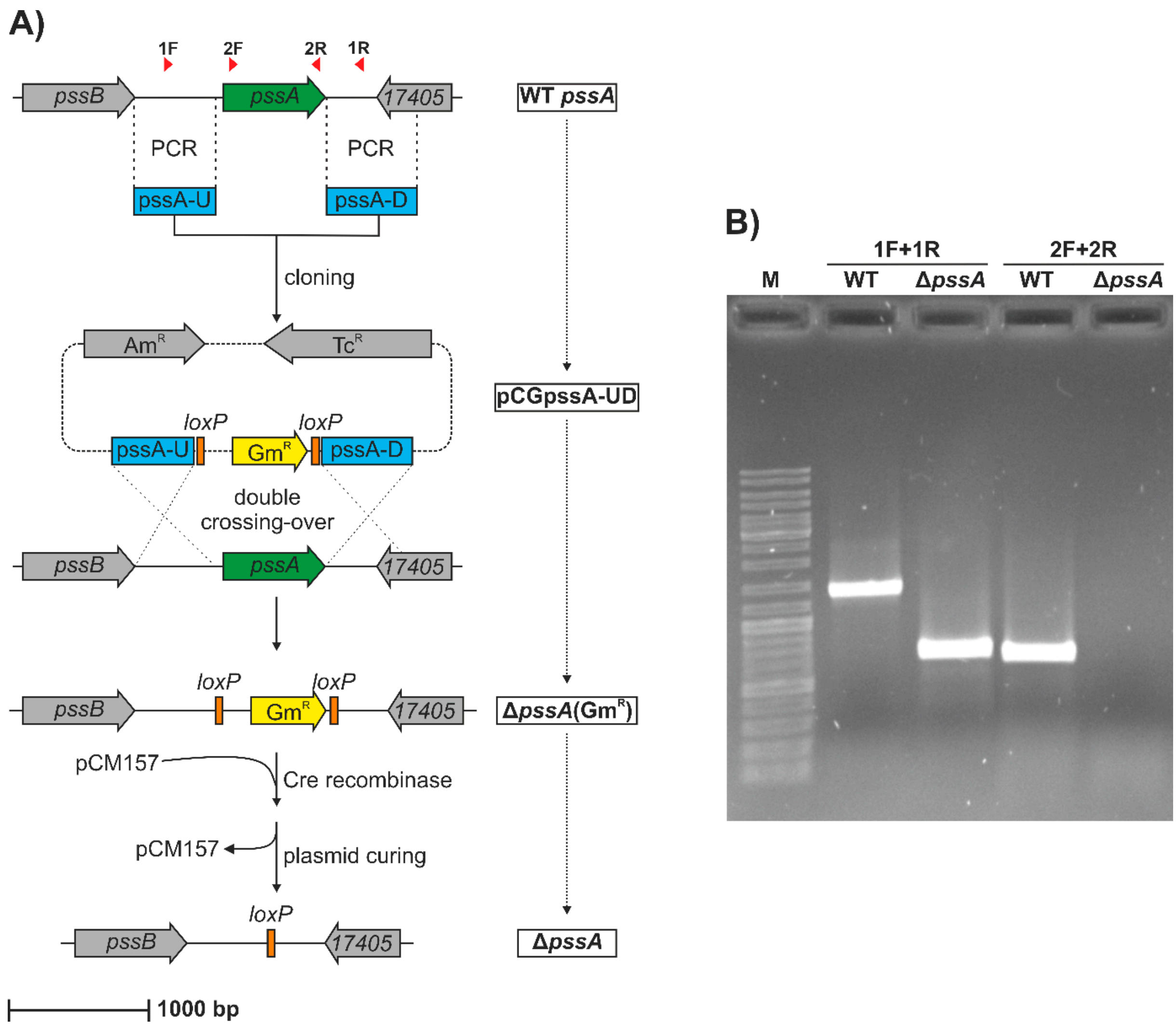

Supplementary Fig. S1. Construction of the RtTA1 Δ*pssA* mutant strain. A) Schematic representation of the construction steps of the RtTA1 Δ*pssA* mutant strain. Details of the mutagenesis and the plasmids used are described in the "Materials and Methods" section. The red arrows at the top of the diagram (named 1F, 1R, 2F, and 2R, respectively) represent the positions of the primers used to confirm successful mutagenesis of *pssA* (B). PCR was performed on gDNA templates isolated from the RtTA1 wild-type (WT) and the Δ*pssA* mutant strains. 1F – pssA-O_Fw primer, 1R – pssA-O_Rv primer, 2F – pssA-I_Fw primer, and 2R – pssA-I_Rv primer (Supplementary Table S2).

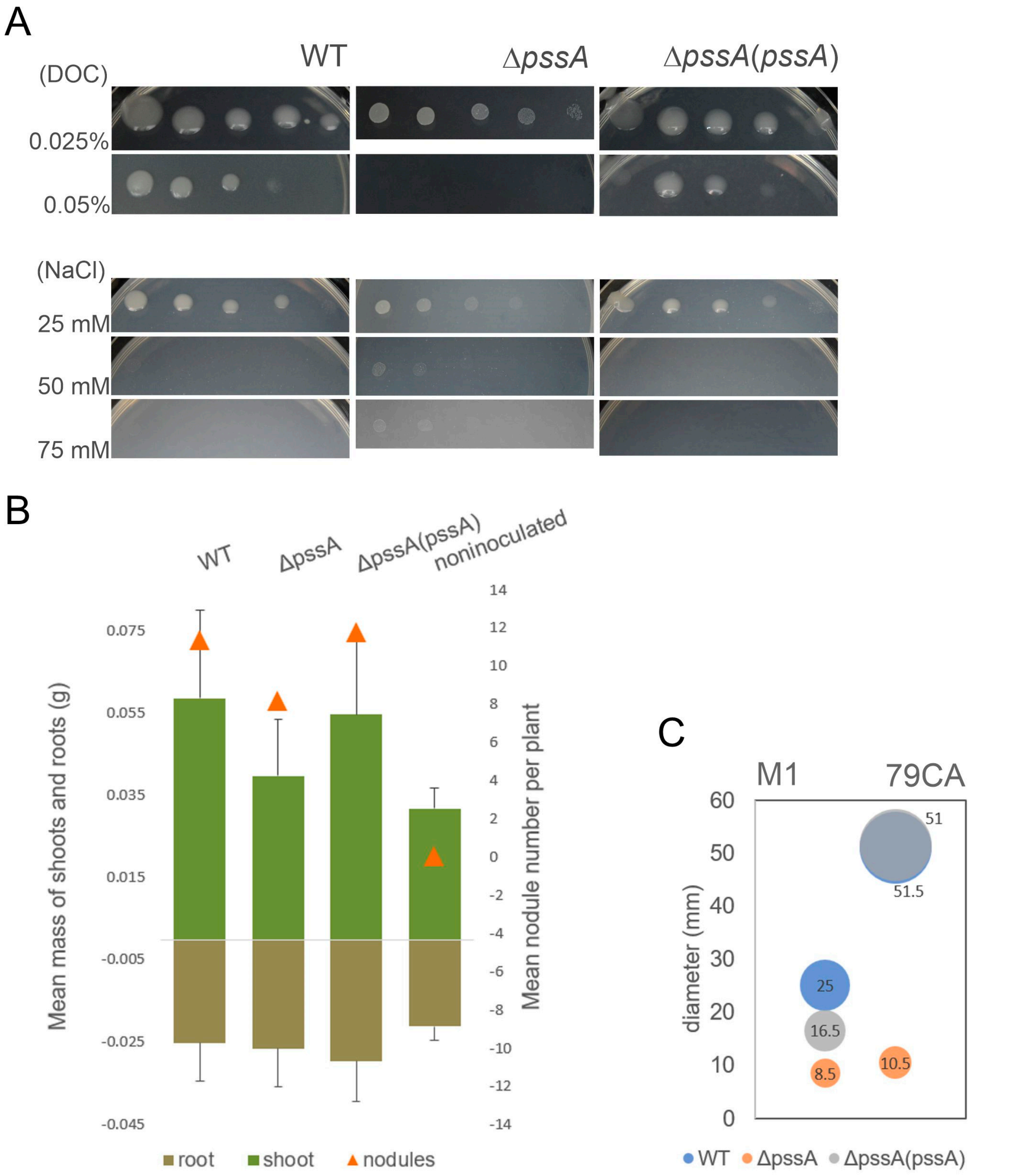

Supplementary Fig. S2. Influence of the *pssA* gene deletion on the selected phenotypic traits of the RtTA1 strain. A) Plate sensitivity tests revealed that *pssA* deletion is associated with increased sensitivity to deoxycholate and slightly decreased sensitivity to excess sodium chloride. B) Plant test confirmed diminished symbiotic performance characteristic for non-EPS-producing rhizobia. C) Swimming motility observed in the semi-solid 0.3%-agar medium was substantially reduced in a case of Δ*pssA* when compared to the WT, both in the minimal M1 and complete 79CA media.

	T25-S	S-T25	T25-E	E-T25	T25-D	D-T25	A-T25	T25-A	T25-C	C-T25	T25-F	F-T25	T25-I	I-T25	T25-J	J-T25	T25-G	G-T25	T25-H	H-T25	T25-P	T25-T	T25-L	T25-P2
S-T18							112.5	117.2																
T18-S							93.4	95.1																
E-T18							98.8	101.8																
T18-E							103.5	95.3																
D-T18							107.5	106.2																
T18-D							100.3	104.2																
A-T18	121.0	125.6	138.5	112.2	134.9	121.1	125.9	**406.1**	97.9	128.2	99.2	107.0	104.5	99.7	101.3	103.9	117.5	96.7	95.6	106.8	107.5	103.5	105.7	104.8
T18-A	98.8	99.6	95.9	106.7	101.3	96.7	**181.9**	97.8	**222.7**	109.3	**184.9**	**216.5**	**241.2**	94.0	**197.6**	139.0	117.8	103.2	93.7	104.6	105.4	103.3	104.8	111.4
C-T18							107.2	**222.9**																
T18-C							98.5	**209.5**																
F-T18							103.6	105.7																
T18-F							103.1	**175.0**																
I-T18							114.8	**623.5**																
T18-I							96.8	**555.5**																
J-T18							100.6	112.2																
T18-J							100.3	**865.6**																
G-T18							100.3	134.5																
T18-G							100.4	105.8																
H-T18							105.2	102.0																
T18-H							102.7	96.6																
T18-P							110.1	95.7																
T-T18							117.0	115.7																
T18-T							108.5	112.0																
L-T18							108.5	119.7																
T18-L							117.5	113.4																
P2-T18							100.0	116.5																
T18-P2							117.3	114.0																

	T25-S	S-T25	T25-E	E-T25	T25-D	D-T25	A-T25	T25-A	T25-C	C-T25	T25-F	F-T25	T25-I	I-T25	T25-J	J-T25	T25-G	G-T25	T25-H	H-T25	T25-P	T25-T	T25-L	T25-P2
S-T18							13.4	9.0																
T18-S							2.7	1.7																
E-T18							3.5	5.6																
T18-E							5.3	8.2																
D-T18							4.0	2.8																
T18-D							3.5	3.4																
A-T18	14.2	14.8	7.4	18.3	18.6	8.3	14.8	44.7	12.6	4.9	3.6	4.0	3.5	2.2	12.2	7.2	10.1	5.4	5.2	1.9	4.0	0.6	0.3	0.8
T18-A	6.8	5.6	2.7	14.4	3.6	4.7	15.1	5.8	16.9	5.4	11.0	12.4	12.6	3.1	24.4	13.0	7.2	5.2	5.6	8.8	0.9	1.1	1.0	1.1
C-T18							11.8	17.6																
T18-C							1.7	8.1																
F-T18							2.1	4.2																
T18-F							2.4	4.8																
I-T18							11.6	29.6																
T18-I							9.7	22.1																
J-T18							1.3	26.9																
T18-J							2.9	56.3																
G-T18							2.9	3.8																
T18-G							4.5	1.7																
H-T18							5.5	4.6																
T18-H							2.2	2.4																
T18-P							0.9	1.0																
T-T18							2.4	0.8																
T18-T							1.2	2.6																
L-T18							4.4	4.8																
T18-L							1.6	0.4																
P2-T18							3.1	1.2																
T18-P2							0.7	6.1																

Supplementary Fig. S3. Activity of β-galactosidase measured in *E. coli* DHM1 strain carrying pairs of T18- and T25-fusion plasmids. Letters A, D, E, etc. in plasmid names correspond with the names of *pss* genes. The position of the letter indicates whether Pss protein constitutes the N- or C-terminal part of the fusion protein. Top: activities presented as mean values of three independent experiments with two technical repeats each; bottom: standard deviations for respective values presented in the top panel. Blue boxes shade significant activities. Negative controls [pUT18(pUT18C) x pKT25(pKNT25)] were equal 90.7 ± 4.9 – 98.9 ± 2.1, and positive control (pUT18zip x pKT25zip) was equal 727.1 ± 43.0 (Miller units).

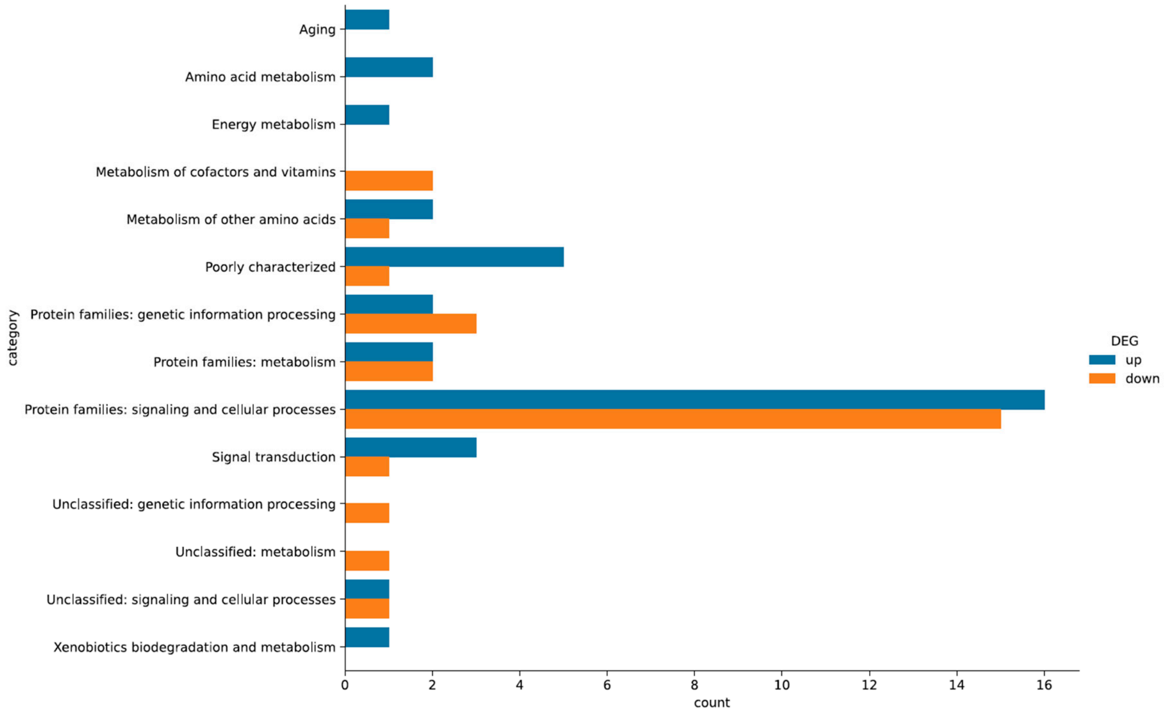

Supplementary Fig. S4. Representation of KEGG pathway categories comprising DEGs of *R. leguminosarum* bv. *trifolii* Δ*pssA* mutant

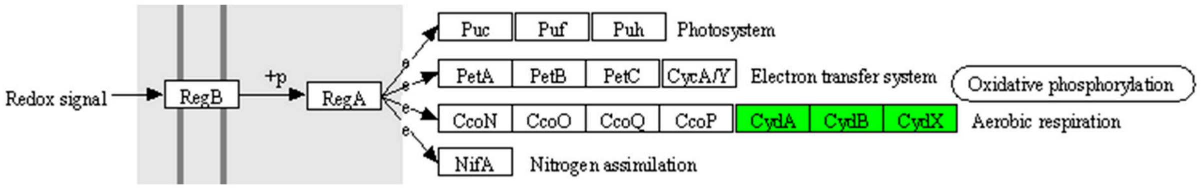

Supplementary Fig. S5. Expression of DEGs involved in oxidative phosphorylation in the Δ*pssA* mutant. The scheme was prepared through the KEGG Mapper Color tool and edited manually. The green boxes correspond to the putative proteins encoded by RLTA1_RS29620 (*cydB*), RLTA1_RS29625, RLTA1_RS29630, and RLTA1_RS29615 (*cydX*) genes upregulated in the Δ*pssA* mutant.

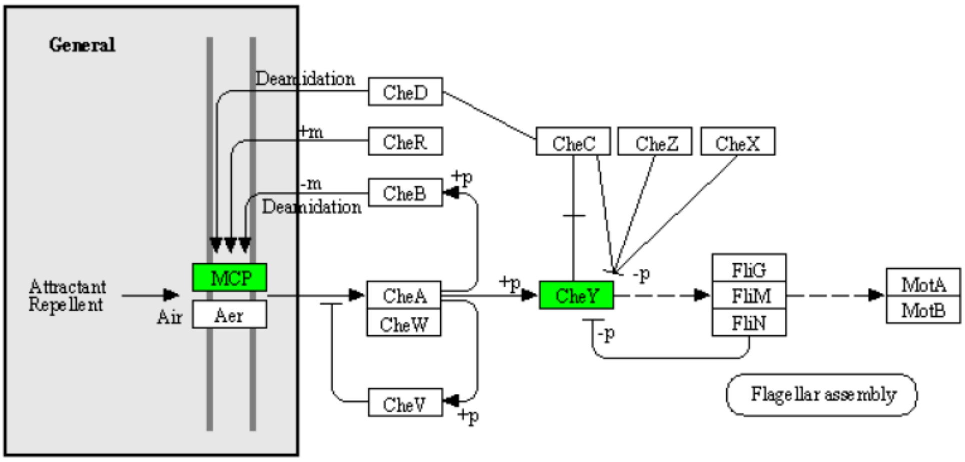

Supplementary Fig. S6. Expression of DEGs involved in chemotaxis and motility in the Δ*pssA* mutant. The scheme was prepared through the KEGG Mapper Color tool and edited manually. The green boxes correspond to the putative proteins encoded by RLTA1_RS13105 (*mcpV*) and RLTA1_RS18655 (*cheY3*) genes upregulated in the Δ*pssA* mutant.

Supplementary File S1. Optimization of high-quality RNA extraction protocol from *Rhizobium leguminosarum*

The isolation of high-quality non-degraded RNA is the first and key step in many molecular biology techniques, including RT-PCR, qPCR, and RNA-seq, which provide valuable information on gene expression, function, and regulation. There are several guidelines for starting RNA to be used in next-generation sequencing (NGS) such as a high ratio of A_{260}/A_{280} (≈2.0) and A_{260}/A_{230} (≥2.0) to measure RNA purity, and a high ratio of 23S rRNA to 16S rRNA (≈1.5) or high RNA Integrity Number (RIN) value (≥7.0) to measure RNA integrity [13, 14]. Determination of RNA integrity by the 23S/16S ratio is a more traditional method but it was suggested that this measure should not be used as the standard for assessing RNA integrity because of its high variability [14]. Currently, RNA quality is routinely judged using automated microfluidic gel electrophoresis platforms and associated software programs.

In this work, we develop and optimize a reproducible protocol for the isolation of high-quality DNA-free total RNA from soil bacteria *Rhizobium leguminosarum* bv. *trifolii* TA1 (RtTA1), which produces large amounts of diverse cell-surface polysaccharides [15]. The performance of five commercially available bacterial total RNA isolation kits was compared, and two methods of cell lysis were tested: enzymatic (with the use of lysozyme) and mechanical. All steps of RNA isolation were performed according to the instructions provided by the kit manufacturers. In each case, total RNA was isolated from the bacterial pellet from 1 ml of log-phase RtTA1 liquid culture in 79CA medium (OD_{600} ≈0.7–0.8; ≈10^9 CFU). In the case of RNA extraction schemes with mechanical lysis, the FastPrep-24 5G homogenizer and the Lysing Matrix B (MP Biomedicals, LLC, Irvine, CA, USA) were used (speed setting of 6.0 m/s for 60 s, room temperature). The following isolation schemes were tested (Table S8):

1A) Total RNA Purification Kit (Norgen Biotek Corp., Thorold, ON, Canada), enzymatic lysis (protocol available at: https://norgenbiotek.com/sites/default/files/resources/PI17200-38%20Total%20RNA%20Purification%20Kit%20Insert.pdf);

1B) Total RNA Purification Kit (Norgen Biotek Corp., Thorold, ON, Canada), mechanical lysis (protocol available at: https://norgenbiotek.com/sites/default/files/resources/App-Note-45-Total-RNA-Isolation-from-Hard-to-Lyse-Bacteria_0.pdf);

2A) Monarch Total RNA Miniprep Kit (New England Biolabs, Inc., Ipswich, MA, USA), enzymatic lysis (protocol available at: https://international.neb.com/protocols/2017/11/08/total-rna-purification-from-tough-to-lyse-bacteria-yeast-plant-etc-samples-using-the-monarch-total-rna-miniprep-kit-neb-t2010);

2B) Monarch Total RNA Miniprep Kit (New England Biolabs, Inc., Ipswich, MA, USA), mechanical lysis (protocol available at: https://international.neb.com/protocols/2017/11/08/total-rna-purification-from-tough-to-lyse-bacteria-yeast-plant-etc-samples-using-the-monarch-total-rna-miniprep-kit-neb-t2010);

3) FastRNA SPIN Kit for Microbes (MP Biomedicals, LLC, Irvine, CA, USA), mechanical lysis (protocol not available on the manufacturer's website, supplied with the purchased kit);

4) Universal RNA Purification Kit (EURx Sp. z o.o., Gdańsk, Poland), enzymatic lysis (protocol available at: https://eurx.com.pl/docs/manuals/en/e3598.pdf);

5) Environmental DNA & RNA Purification Kit (EURx Sp. z o.o., Gdańsk, Poland), both enzymatic and mechanical lysis (protocol available at: https://eurx.com.pl/docs/manuals/en/e3572.pdf).

Table S8. Summary of total RNA isolation from RtTA1 using various isolation schemes

Isolation scheme	RNA isolation kit	Method of cell lysis	Efficiency [µg][a]	Purity		RIN	23S/16S rRNA ratio	Representative electropherogram
				A_{260}/A_{280}	A_{260}/A_{230}			
**1A**	Total RNA Purification Kit	enzymatic	2.4	2.1	1.5	8.4	1.3	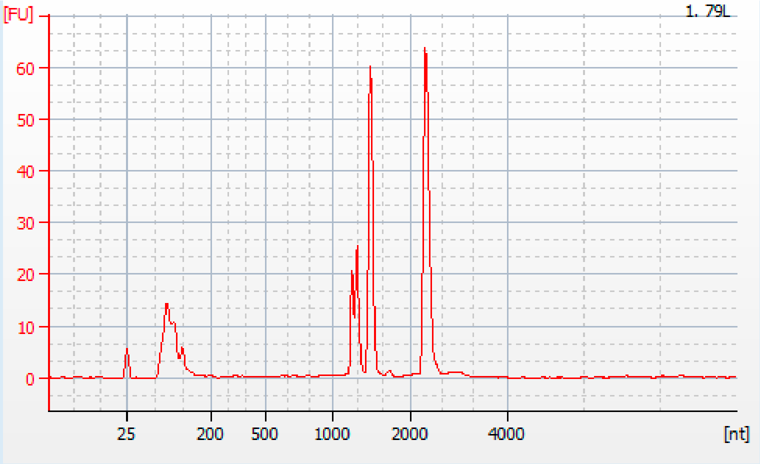
**1B**		mechanical	2.1	2.1	1.0	6.3	1.0	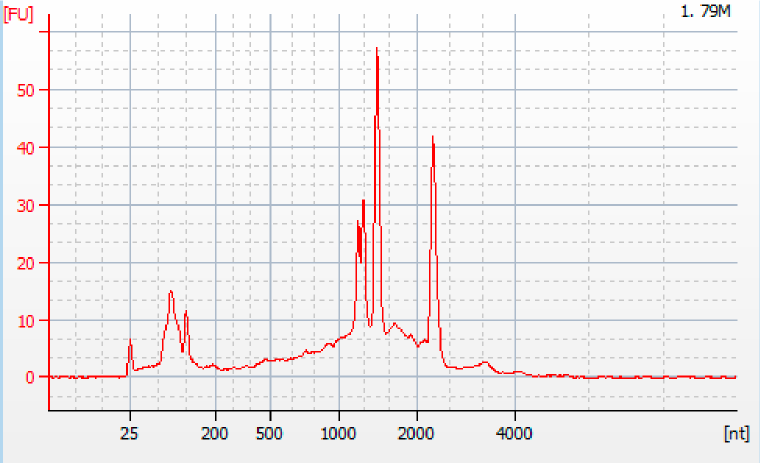

**2A**	Monarch Total RNA Miniprep Kit	enzymatic	2.8	2.1	2.4	8.4	1.4	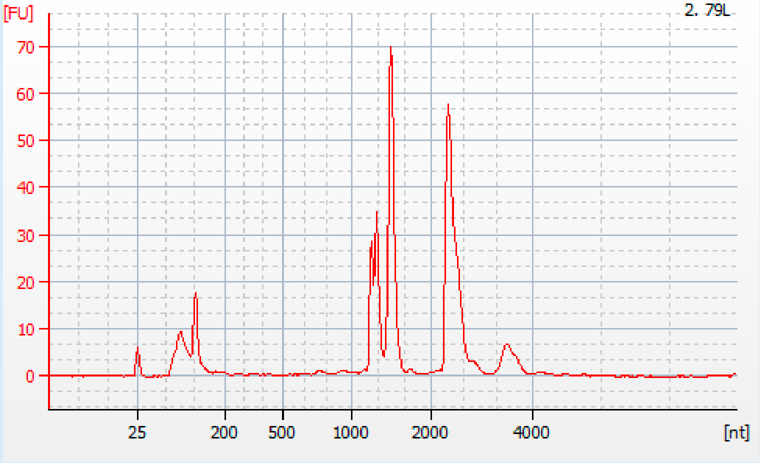
**2B**		mechanical	1.3	1.9	2.5	3	0.8	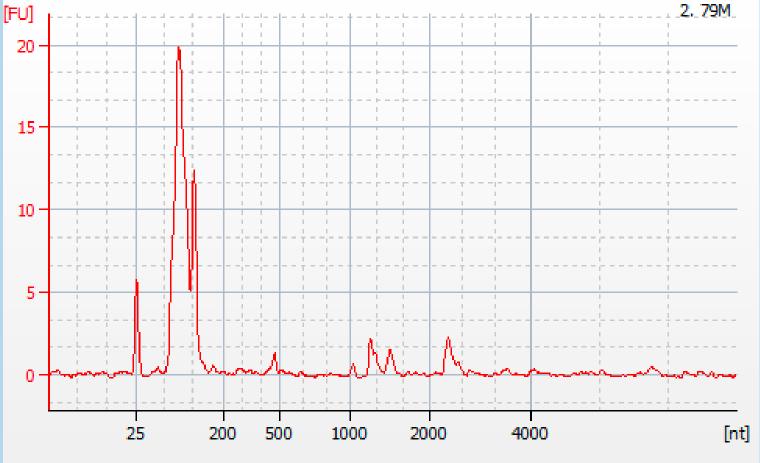
**3**	FastRNA SPIN Kit for Microbes	mechanical	2.3	2.0	2.5	8.0	1.0	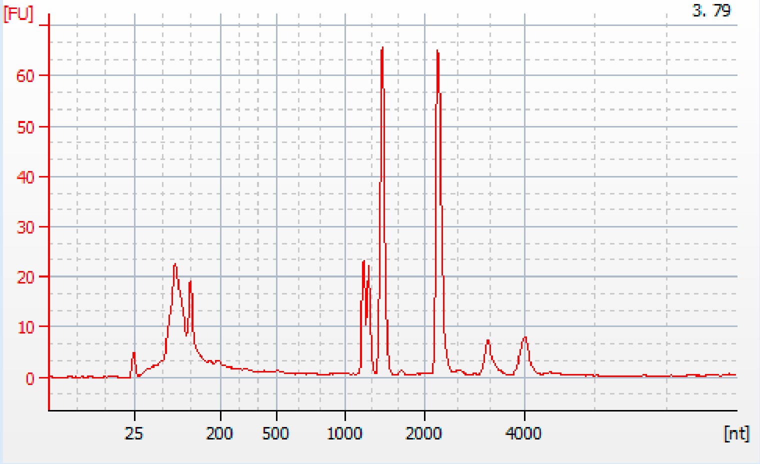

**4**	GeneMATRIX Universal RNA Purification Kit	enzymatic	3.5	2.1	2.4	8.7	1.2	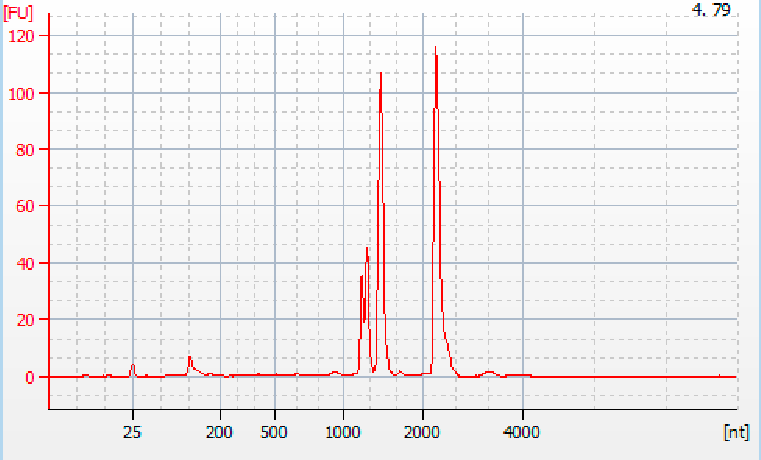
**5**	GeneMATRIX Environmental DNA & RNA Purification Kit	both enzymatic and mechanical	4	2.2	2.4	6.9	1.4	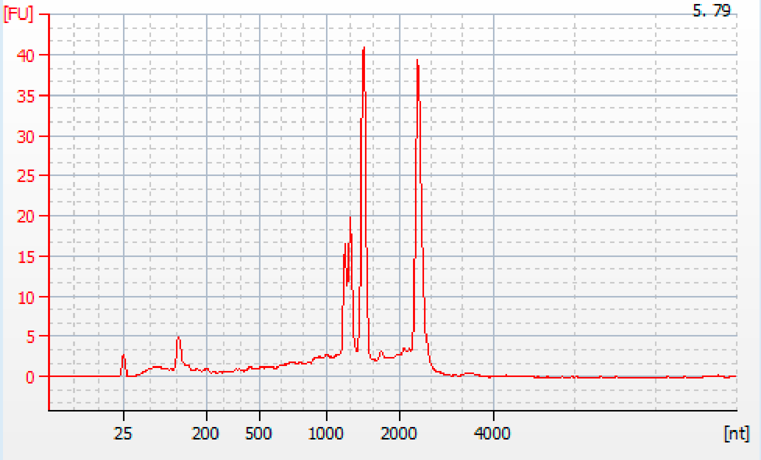

[a] RNA isolation efficiency from the bacterial pellet from 1 ml of log-phase RtTA1 liquid culture in 79CA medium (OD_{600} ≈0.7–0.8; $\approx 10^9$ CFU)

For each isolation scheme, the total RNA was eluted with 50 µl of sterile nuclease-free water (Thermo Fisher Scientific, Waltham, MA, USA). The quantity and quality of RNA were checked spectrophotometrically (Synergy H1 reader, Agilent Technologies, Inc., Santa Clara, CA, USA), fluorometrically (Qubit 2.0 Fluorometer with Qubit RNA High Sensitivity (HS) Assay Kit, Thermo Fisher Scientific, Waltham, MA, USA), in microcapillary electrophoresis (2100 Bioanalyzer Instrument with RNA 6000 Nano Kit, Agilent Technologies, Inc., Santa Clara, CA, USA), and in the PCR reaction. Table S8 summarizes the efficiency of total RNA isolation from RtTA1 cells using five commercial RNA isolation kits and two types of lysis. The summary in Table S8 is the result of two independent experiments for each of the RNA isolation schemes. The largest amounts of RNA (>3 µg) were isolated using protocols 4 and 5. Almost in all tested isolation variants, the A_{260}/A_{280} ratio was around 2.0 and the A_{260}/A_{230} ratio was ≥2.0, indicating the high purity of the RNA preparations. RNA integrity as measured by the RIN index was the highest (≥8.4) for the isolation protocols with enzymatic lysis (schemes 1A, 2A, and 4). The ratios of 23S rRNA to 16S rRNA were also the highest in these isolation variants. It should be noted that in the case of total RNA isolation from RtTA1, both this parameter and the RIN value are underestimated. The Bioanalyzer software is based on limited data sets of model organisms and performs data interpretation with the assumption that prokaryotic rRNA molecules are eluted in two unique peaks corresponding to 23S and 16S molecules [13]. However, some microorganisms carry fragmentation sites in their rRNA structural genes which are post-transcriptionally cleaved during rRNA maturation and ribosome formation [16]. In such cases, the 23S and 16S ribosomal RNA components elute in multiple peaks, generating false quality results and lower 23S to 16S rRNA ratios. *R. leguminosarum* possesses two processing sites in its 23S rRNA (precursor molecule) of 2.9 kb, giving rise to 5.8S-like rRNA (approx. 130 nt) and two approx. 1.3 kb rRNA fragments [17]. The reduced ratios between 23S and 16S rRNA and additional rRNA peaks are clearly visible on the electropherograms of isolated RNA (Table S8). Overall, the best reproducibility, quantity (≈3.5 µg total RNA from ≈10^9 CFU), and RNA quality (RIN value of 8.7 and intact RNA visible on electropherograms) were obtained using scheme 4 (Table S8).

Genomic DNA contamination was then checked by PCR using primers targeting the *repC/a* (located on the pRleTA1a megaplasmid, 167 bp product) and *pssGF* (chromosomally-located, 277 bp product) genes (Supplementary Table S2). In the case of all RNA samples, the PCR reaction showed distinct bands of the assumed size, indicating that RNA is contaminated with DNA (Supplementary Figure S7). This was especially surprising for isolation schemes 2 and 4, which offered on-column digestion of DNA during the RNA isolation procedure.

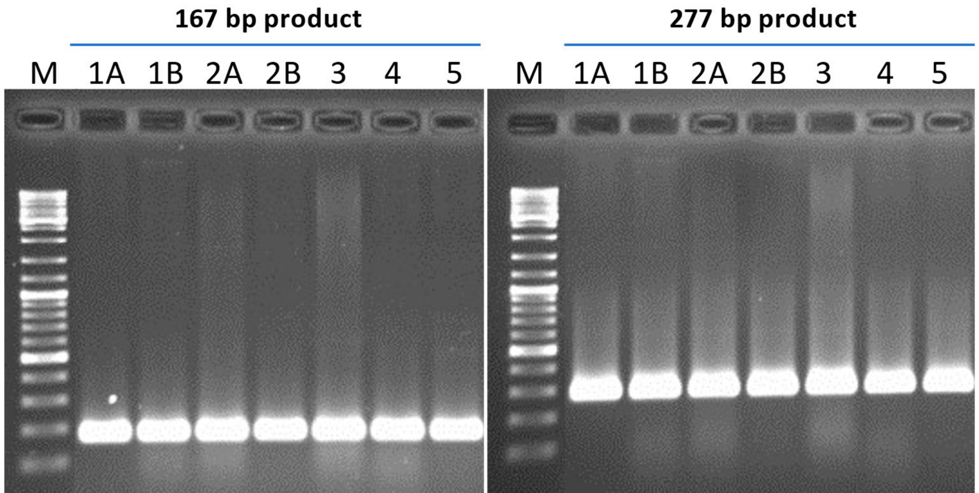

Fig. S7. PCR reaction on the template of total RNA obtained with different isolation schemes. Visible amplicons indicate contamination of RNA isolates with the genomic DNA

Optimization of gDNA elimination was performed for RNA isolates obtained according to scheme 4. To eliminate contaminating DNA from RNA preparations, the TURBO DNA-free Kit (Thermo Fisher Scientific, Waltham, MA, USA) was used according to the rigorous DNase treatment (protocol available at: https://tools.thermofisher.com/content/sfs/manuals/cms_055740.pdf) with a slight modification. Namely, we performed three rounds of DNase (2 U) treatment for 30 min at 37°C (6 U of DNase and 90 min of digestion in total). The purified RNA was then used as a template in a control PCR reaction and re-checked for quantity and quality. Residual DNA was not detectable by PCR after 35 cycles, demonstrating the effective elimination of gDNA contamination from RtTA1 RNA samples (Supplementary Figure S8). There was also no evidence of a negative impact of the gDNA elimination step on the quantity and quality of the RNA preparation (except for a slight decrease in A_{260}/A_{230} ratio to 1.9). The developed protocol was used to isolate RNA for high-throughput transcriptomic analyses.

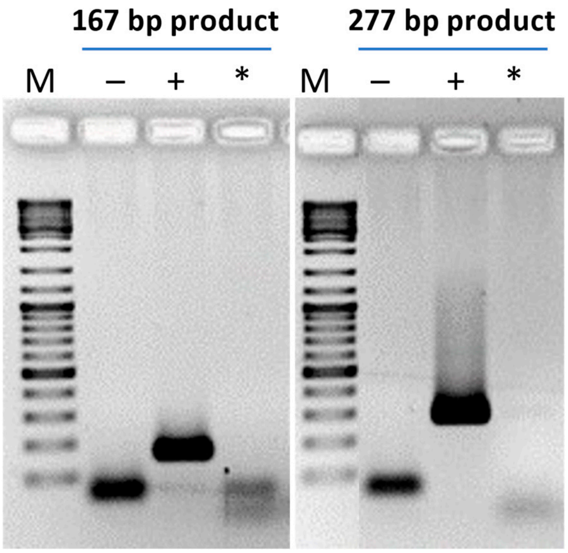

Fig. S8. PCR reaction confirming the elimination of gDNA from the total RNA preparation. The symbols mean: "–" control reaction without template DNA, "+" control reaction with RtTA1 gDNA, and "*" reaction with RNA preparation subjected to DNA elimination
